# Aggressive uveal melanoma displays a high degree of centrosome amplification, opening the door to therapeutic intervention

**DOI:** 10.1002/cjp2.272

**Published:** 2022-04-26

**Authors:** Dorota Sabat‐Pośpiech, Kim Fabian‐Kolpanowicz, Helen Kalirai, Natalie Kipling, Sarah E Coupland, Judy M Coulson, Andrew B Fielding

**Affiliations:** ^1^ Molecular Physiology and Cell Signalling, Institute of Systems Molecular & Integrative Biology University of Liverpool Liverpool UK; ^2^ Molecular and Clinical Cancer Medicine, Institute of Systems Molecular & Integrative Biology University of Liverpool Liverpool UK; ^3^ Biomedical and Life Sciences, Faculty of Health and Medicine Lancaster University Lancaster UK

**Keywords:** uveal melanoma, centrosome amplification, centrosome clustering, pericentriolar matrix, mitotic spindle, monosomy 3, primary, metastatic, proliferation

## Abstract

Uveal melanoma (UM) is the most common intraocular cancer in adults. Whilst treatment of primary UM (PUM) is often successful, around 50% of patients develop metastatic disease with poor outcomes, linked to chromosome 3 loss (monosomy 3, M3). Advances in understanding UM cell biology may indicate new therapeutic options. We report that UM exhibits centrosome abnormalities, which in other cancers are associated with increased invasiveness and worse prognosis, but also represent a potential Achilles' heel for cancer‐specific therapeutics. Analysis of 75 PUM patient samples revealed both higher centrosome numbers and an increase in centrosomes with enlarged pericentriolar matrix (PCM) compared to surrounding normal tissue, both indicative of centrosome amplification. The PCM phenotype was significantly associated with M3 (*t*‐test, *p* < 0.01). Centrosomes naturally enlarge as cells approach mitosis; however, whilst UM with higher mitotic scores had enlarged PCM regardless of genetic status, the PCM phenotype remained significantly associated with M3 in UM with low mitotic scores (ANOVA, *p* = 0.021) suggesting that this is independent of proliferation. Phenotypic analysis of patient‐derived cultures and established UM lines revealed comparable levels of centrosome amplification in PUM cells to archetypal triple‐negative breast cancer cell lines, whilst metastatic UM (MUM) cell lines had even higher levels. Importantly, many UM cells also exhibit centrosome clustering, a common strategy employed by other cancer cells with centrosome amplification to survive cell division. As UM samples with M3 display centrosome abnormalities indicative of amplification, this phenotype may contribute to the development of MUM, suggesting that centrosome de‐clustering drugs may provide a novel therapeutic approach.

## Introduction

Uveal melanoma (UM) is the most common adult primary intraocular malignancy. It is a rare disease with an estimated incidence of 3–9 cases per 1,000,000 people per year in Europe [1, 2]. UM is characterised by specific chromosomal alterations, which affect disease prognosis. The most common alteration is the loss of one copy of chromosome 3 (monosomy 3, M3), which is observed in over 50% of cases and associated with poor prognosis [3, 4, 5]. Other frequent chromosomal abnormalities involve chromosomes 1, 6, and 8 [5, 6, 7, 8, 9]. Amongst M3 cases, 60–92% also exhibit polysomy 8q, and their co‐occurrence is associated with worse prognosis [5, 10]. In contrast, chromosome 6p gain, with an absence of other abnormalities, is associated with good prognosis [9, 11].

The available treatment options for primary UM (PUM) include surgery (local tumour resection, endoresection, enucleation) or radiotherapy (proton beam irradiation or plaque brachytherapy) [12, 13, 14]. However, despite good control of PUM, around 50% of patients develop metastases, usually in the liver (85%), lungs (17%), and bone (16%) [15]. At present, there is no curative treatment available for metastatic UM (MUM), and therefore there is an urgent need to better understand UM biology and to explore new targeted treatments, including via clinical trials [16, 17].

Centrosome amplification is a recognised hallmark of cancer. High levels of centrosome aberrations are often associated with aggressive disease [18] and centrosome amplification can induce oncogenic phenotypes including aneuploidy [19] and invasiveness [20, 21]. Centrosomes are small organelles consisting of a pair of centrioles surrounded by a pericentriolar matrix (PCM) [22, 23]. In normal cells, centrosomes are duplicated once per cell cycle during S‐phase [24] so that two mature centrosomes are present in mitotic cells, which nucleate the minus ends of microtubules to form the bipolar mitotic spindle [25]. However, cancer cells across a diverse range of tumour types display centrosome amplification, where cells contain more than two centrosomes [26, 27]. This is often accompanied by structural centrosome aberrations, including increased size of the PCM [18, 27, 28] and increased length of centrioles [29], both of which are strongly linked to centrosome amplification. Overly elongated centrioles may fragment and mature into extra centrioles and are, therefore, a mechanism driving centrosome amplification in cancer [29]. Abnormally enlarged areas of PCM are frequently observed to contain supernumerary centrioles [18, 27]. Indeed, measurement of the area of PCM has been established as a clinically relevant method to score functional centrosome aberrations in cancer [18].

Centrosome amplification presents a dichotomy, as it promotes a range of oncogenic phenotypes, including those associated with metastasis, but is also a targetable feature of cancer (reviewed in Ref. [30]). Cancer cells must manage their supernumerary centrosomes during mitosis, most often by centrosome clustering, to allow successful bipolar division and cell proliferation [31, 32]. If this process is disrupted, cells form multipolar mitoses, which almost inevitably lead to cell death. Therefore, there is considerable ongoing research into proteins required for centrosome clustering, or other centrosome amplification coping mechanisms, as cancer‐specific therapeutic targets. Functionally, the increased frequency of centrosomes with large areas of PCM staining correlates strongly with sensitivity to depletion of the centrosome clustering protein Kinesin Family Member C1 (KIFC1) [18]. Small molecule inhibitors are being developed for KIFC1 and other targets linked to centrosome abnormalities (reviewed in Ref. [33]).

Here, we investigated centrosome status in UM for the first time. We find that centrosome amplification and centrosomes with enlarged PCM are common in PUM tissue samples, where PCM enlargement is associated with more highly proliferative tumours, M3, and worse prognosis. We also show that centrosome amplification is prevalent in low passage patient‐derived PUM cells and established UM cell lines, with the highest frequency and greatest severity of centrosome amplification in cell lines derived from MUM. Centrosome clustering is evident in both PUM and MUM cell lines, suggesting centrosome de‐clustering drugs as a potential novel therapeutic approach.

## Materials and methods

### Tissue samples

UM samples were obtained from the Health Research Authority approved Ocular Oncology Biobank (REC Ref 21/NW/0139) under project‐specific ethical approval 15/SC/0611. Samples for immunohistochemistry (IHC) were formalin‐fixed paraffin‐embedded (FFPE) primary enucleation specimens (i.e. treatment naïve), collected between 2010 and 2016, and previously worked up morphologically as per diagnostic standard procedures, with haematoxylin and eosin (H&E) staining. Of the 75 samples included in the study, 65 contained both tumour and the inner nuclear layer of the retina (control area) for analysis, while in the remaining 10 cases only tumour was available. PUM cultures (*n* = 4) were established as previously described [34] and grown in 8‐well chamber slides until they were 60–70% confluent. All experiments were conducted according to the Declaration of Helsinki and following all local policies and procedures for work with human material.

### Immunohistochemistry

Staining procedures were performed using the BOND‐RXm autostainer (Leica Biosystems, Milton Keynes, UK) according to the manufacturer's instruction using J protocol, Refine Red Detection kit (RED, DS9390), and antigen retrieval solution (pH 9.0). A pericentrin antibody (Abcam, Cambridge, UK, ab4448) was used at 1:1,000 dilution and Ki‐67 antibody (Novacastra, Leica Biosystems, Wetzlar, Germany, NCL‐L‐Ki67‐MM1) was used at 1:100 dilution. Sections were counterstained with haematoxylin prior to dehydration through a series of ethanol (*n* = 3) (Fisher Scientific UK Ltd, Loughborough, UK, M/4450/17) and xylene (*n* = 3) (Fisher, X/0100/17). Subsequently, samples were mounted using DPX mountant (Merck Life Science UK Ltd, Dorset, UK) with coverslips and left to dry overnight before imaging. Ki‐67 analysis was undertaken, as previously described [35]; namely, the percentage of Ki‐67‐positive UM cells was determined by SEC for each tumour. For pericentrin analysis, samples were scanned using the Roche Ventana DP200 slide scanner (Roche Diagnostics Ltd, West Sussex, UK). Images were analysed using Aperio ImageScope [v12.3.2.8013] and ImageJ [2.0.0‐rc‐54/1.51h] software. In brief, for each sample, three separate ×40 fields of view were selected from the tumour area and inner nuclear layer of the retina (control). Colour deconvolution was performed between RED and haematoxylin signals, and images were converted into binary data. The number of visible cell nuclei, as well as the number and surface area of visible pericentriolar clouds were calculated using automatic scoring. The centrosome score was calculated by dividing the number of pericentrin‐positive foci by the number of nuclei.

### Tissue culture

All tissue culture reagents were supplied by Gibco/Thermo Fisher Scientific (Loughborough, UK) unless otherwise stated. PUM cell cultures (*n* = 4) were collected from the Ocular Oncology Biobank under the project specific ethics detailed above. The UM cell lines Mel270, OMM2.3, OMM2.5, 92.1, MP46, and MM66 originated from the labs where they were established [36, 37, 38, 39] and were cultured in RPMI media supplemented with 10% foetal calf serum, 50 IU/ml penicillin, and 50 μg/ml streptomycin. BT549 cells (ATCC, Manassas, VA, USA) were cultured in RPMI supplemented with 10% foetal bovine serum (FBS) and 72 ng/ml insulin (Sigma‐Aldrich, St Louis, MO, USA). hTert‐HME1 (ATCC) cells were cultured in MEGM plus supplements, according to ATCC guidelines (MEGM BulletKit, CC‐3150, Lonza, Manchester, UK, without the addition of gentamycin‐amphotericin B). MCF10A cells were cultured in DMEM/F‐12 plus 10% FBS, 5% horse serum, 20 ng/ml EGF, 100 ng/ml cholera toxin, 500 ng/ml hydrocortisone, and 10 μg/ml insulin. Cells were cultured at 37°C with 5% CO_2_ and passaged before cultures became confluent. Cells were regularly screened for Mycoplasma using the MycoAlertTM Mycoplasma detection kit (LT07‐318, Lonza) and were Mycoplasma‐free at the time of the experiments. Cell line authentication was performed by Eurofins' short tandem repeat profiling service.

### Immunofluorescence staining

Cells in chamber slides or 1 cm glass coverslips placed in 12‐well culture plates were cultured for 2 days (Mel270: 140,000 cells; 70,000 cells for all other cell lines). Cells were fixed with ice‐cold methanol for 15 min at −20°C. Subsequent incubations were performed at room temperature for 1 h. Antibodies were diluted in 5% goat serum in PBS. Coverslips were blocked in 5% goat serum (Gibco/Thermo Fisher Scientific) in PBS and then incubated with primary antibody mixes: either rabbit anti‐pericentrin (Abcam ab4448) and mouse anti‐alpha‐tubulin (Sigma‐Aldrich T6199), or rabbit anti‐pericentrin (Abcam ab4448), mouse anti‐Centrin (Millipore, Burlington, MA, USA, 04‐1624), and rat anti‐alpha‐tubulin (Millipore MAB1864). Coverslips were washed with PBS before incubation with secondary antibody mixes whilst protected from light: goat anti‐rabbit Alexa Fluor Plus 555 (Invitrogen, Waltham, MA, USA, A32732), goat anti‐mouse Alexa Fluor Plus 488 (Invitrogen A32723), and, where necessary, goat anti‐rat Alexa Fluor 647 (Invitrogen A21247; all 1/500). Coverslips were washed with PBS before mounting in Mowiol containing 4′,6‐diamidino‐2‐phenylindole (DAPI, Sigma‐Aldrich) at 1 μg/ml. Slides were stored at 4 °C, protected from light.

### Imaging of immunofluorescence

Slides were imaged using a Plan‐Apochromat 40×/1.4 Oil DIC M27 objective on a Zeiss LSM880 confocal microscope (Carl Zeiss Ltd., Oberkochen, Germany) with Zen software (version 14). For the analysis of interphase cells, large fields of view were acquired and the pericentrin stain was used to identify centrosomes. For the analysis of the patient‐matched Mel270, OMM2.5, and OMM2.3 cells in Figure 4, slides were randomised for blind analysis. Mitotic cells were identified in a methodical manner, scanning a coverslip from right to left, top to bottom, and Z‐stacks covering the depth of mitotic cells were acquired. Zen software was used to create maximum‐intensity projections of the Z‐stacks and images were analysed in ImageJ using the merged pericentrin/centrin channels to identify centrosomes.

## Results

### Centrosome amplification is evident by two criteria in PUM tissue sections

**Figure 1:**
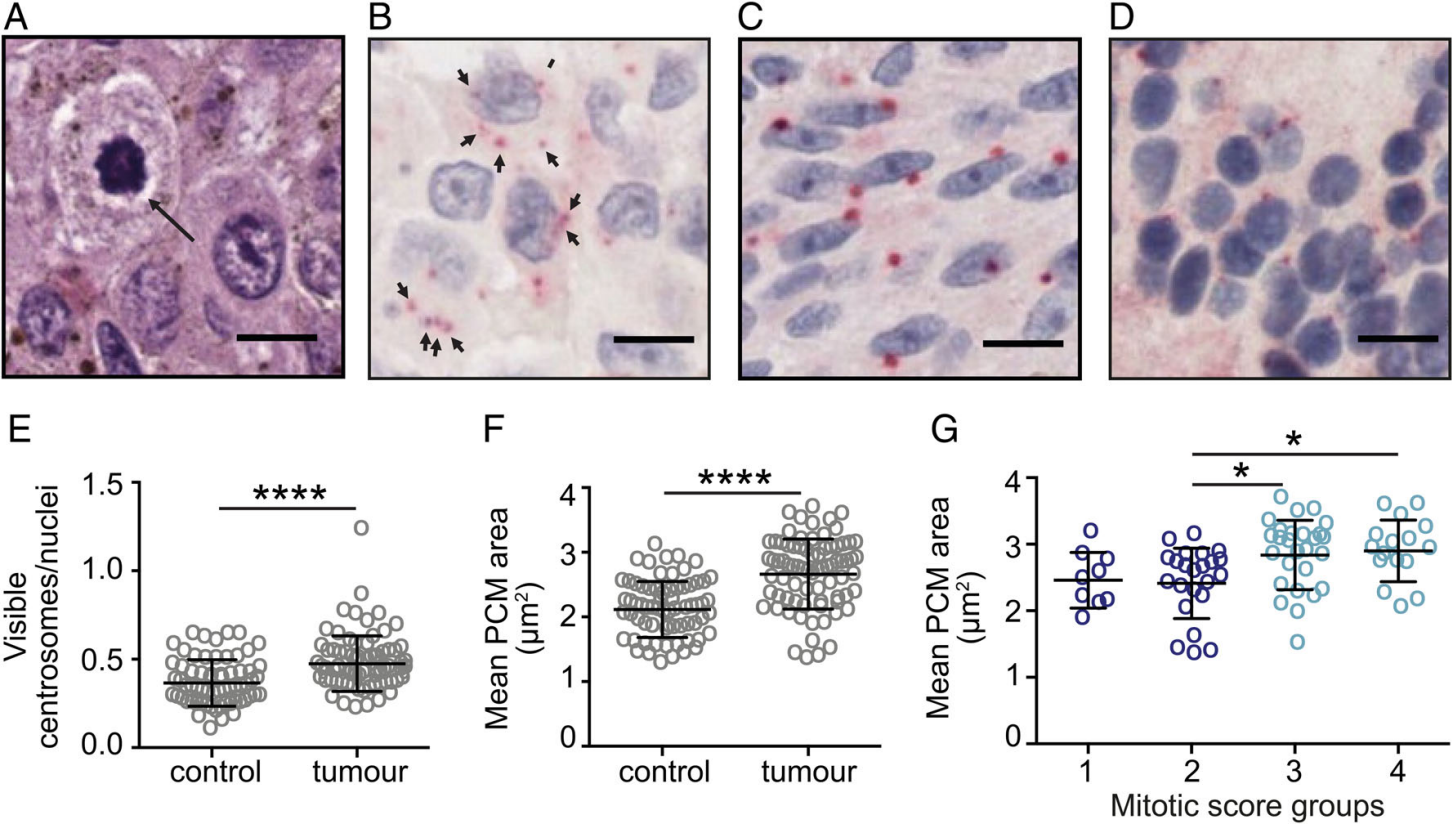
Abnormal mitotic phenotypes and centrosome abnormalities are present in UM. (A) PUM H&E staining showing a multipolar mitosis. Scale bar 10 μm. (B–G) PCM size and centrosome number were measured in a cohort of 75 FFPE enucleation samples. *N* > 100 centrosomes were analysed per sample in both tumour and control regions of sections. Cell nuclei (blue) were stained with haematoxylin, and centrosomes (red) were visualised using anti‐pericentrin antibody and RED chromogen. Scale bar 10 μm. (B) Example image of UM showing cells with amplified centrosomes (marked with arrows). Example images of (C) UM with enlarged PCM and (D) adjacent retina used as the control for PCM size. (E, F) Scoring for (E) the number of visible pericentrin‐positive foci relative to the number of visible nuclei, or (F) mean PCM size, in control (*n* = 65) versus tumour (*n* = 75) regions. Error bars indicate standard deviation around the mean; unpaired two‐tailed *t*‐test, ****p < 0.0001. (G) Comparison of mean PCM size in tumour regions categorised into mitotic score groups: 1 (0–1 mitoses per ×40 field of view), 2 (2–3 mitoses), 3 (4–7 mitoses), and 4 (>7 mitoses). Ordinary one‐way ANOVA with Tukey *post hoc* test; significant differences between groups are indicated, *p < 0.05.

Abnormal mitotic figures were present in some H&E‐stained treatment‐naïve PUM enucleation samples (Figure 1A), suggestive of multipolar mitoses typically caused by supernumerary centrosomes. As centrosome amplification had not previously been reported in UM, but could be a potentially valuable therapeutic target, we investigated further. FFPE sections of 75 enucleated PUM (Table 1) were stained for pericentrin, a well‐validated PCM marker. An example section illustrating the areas scored for centrosomes is shown in supplementary material, Figure S1A. The PUM displayed significantly higher numbers of pericentrin foci per nucleus than control areas (Figure 1B,D,E; *p* < 0.0001), indicative of centrosome amplification.

**Table 1:** Patient characteristics for PUM samples analysed in Figures 1 and 2

Patient features	Total	%
Median age at primary treatment (range), years	63 (39–90)	
Gender		
Male	48	64
Female	27	36
Median largest basal diameter (range), mm	15.7 (5.9–26)	
Median ultrasound height (range), mm	8.4 (1.0–18.3)	
Ciliary body involvement		
No	51	68
Yes	24	32
Extraocular melanoma		
No	65	87
Yes	10	13
Epithelioid cells present		
No	40	53
Yes	35	47
Closed PAS+ loops present		
No	30	40
Yes	45	60
Median mitotic count (range)	4.0 (1–38)	
Chromosome 1p		
Normal	49	65
Loss	18	24
Unclassified	8	11
Chromosome 3		
Normal	35	47
Loss	40	53
Chromosome 6p		
Normal	48	64
Gain	26	35
Unclassified	1	1
Chromosome 6q		
Normal	51	68
Loss	13	17
Gain	5	7
Unclassified	6	8
Chromosome 8p		
Normal	53	70
Loss	11	15
Gain	5	7
Unclassified	6	8
Chromosome 8q		
Normal	41	55
Gain	34	45
Median follow‐up time (range), months	70 (0–122)	
Status		
Alive	42	56
Dead	33	44
Cause of death		
Metastatic melanoma	17	52
Other	6	18
Unknown	10	30

Although this result was clear, it is inherently difficult to score centrosomes in these thin tumour sections that do not span the complete cell depth, as the nucleus and centrosomes from a single cell may not be present in the same plane. This is evident from the mean centrosome/nuclei scores, which were less than 1 in both control and tumour samples (Figure 1E). A clinically applicable method for scoring functionally relevant centrosome aberrations by IHC, based on the area of pericentrin stained foci, was recently developed and validated in breast cancer tissues and cell line FFPE sectional pellets [18]. We used this method, referred to henceforth as mean PCM area, as a complementary measure of centrosome abnormalities in our PUM sample set. The mean PCM area was significantly greater in PUM cells compared to adjacent normal cells (Figure 1C,D,F; *p* < 0.0001). A potential confounding factor of this method is that cells undergo centrosome maturation and recruit more PCM as they approach mitosis; therefore, rapid tumour proliferation could lead to a higher proportion of cells with larger centrosomes. Available data for the cell proliferation marker Ki67 [40] positively correlated with mitotic score (supplementary material, Figure S1B; *p* < 0.0001), which we used as a measure of actively proliferating cells. Overall, the mean PCM area in tumour regions showed a moderate correlation with mitotic score (supplementary material, Figure S1C; *p* < 0.0001), and PUM in high mitotic score groups displayed significantly greater mean PCM area compared to tumours in low mitotic score groups (Figure 1G; *p* < 0.05). Therefore, we could not exclude the possibility that greater PCM area in PUM compared to control samples may reflect higher proliferation.

### PUM with M3 show higher levels of centrosome aberrations independent of mitotic score

**Figure 2:**
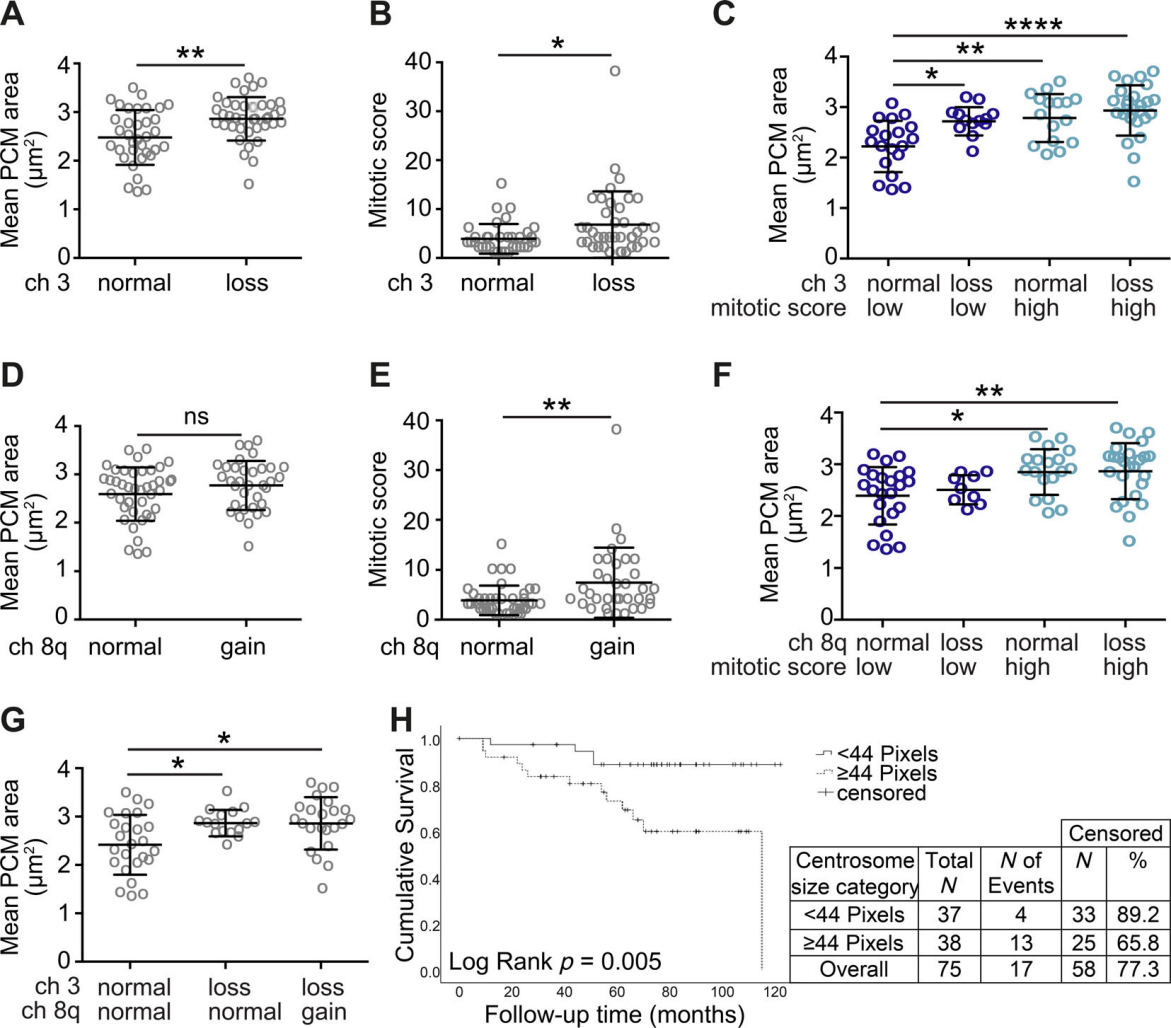
Mean PCM area in UM is influenced by genetic background independent of mitotic score. (A, B) Comparison of (A) mean PCM area and (B) mitotic score in UM tumours with different chromosome 3 status: normal (*n* = 35) versus loss (M3, *n* = 40). Unpaired two‐tailed *t*‐test, *p < 0.05, **p < 0.01. (C) Comparison of mean PCM area in UM tumours categorised by chromosome 3 status and mitotic score. Low mitotic score: groups 1 and 2 (0–3 mitoses). High mitotic score: groups 3 and 4 (≥4 mitoses). Group sizes: normal low (*n* = 19), loss low (*n* = 13), normal high (*n* = 16), loss high (*n* = 27). Ordinary one‐way ANOVA with Tukey *post hoc* test, significant comparisons between groups are indicated, *p < 0.05, **p < 0.01, ****p < 0.0001. (D, E) Comparison of (D) mean PCM area and (E) mitotic score in UM tumours with different chromosome 8q status: normal (*n* = 41) versus gain (*n* = 34). Unpaired two‐tailed *t*‐test, ns – not significant, **p < 0.01. (F) Comparison of mean PCM area in UM tumours categorised by chromosome 8q status and mitotic score. Low and high mitotic score groups as in (C). Group sizes: normal low (*n* = 23), gain low (*n* = 9), normal high (*n* = 18), gain high (*n* = 25). Ordinary one‐way ANOVA with Tukey *post hoc* test, significant comparisons between groups are indicated, *p < 0.05, **p < 0.01. (G) Comparison of mean PCM area in tumours categorised by both chromosome 3 and chromosome 8q status. Group sizes: chromosome 3 and 8q normal (*n* = 25), 3 loss and 8q normal (*n* = 16), 3 loss and 8q gain (*n* = 24). Ordinary one‐way ANOVA with Tukey *post hoc* test, significant differences between groups are indicated, *p < 0.05. (H) Kaplan–Meier curve demonstrating worse survival in the cohort of UM with larger centrosomes (*p* = 0.005).

In UM, M3 is the most common chromosomal alteration and strongest predictor of metastasis and poor survival [4, 5]. Therefore, we investigated whether centrosome aberrations were linked to M3 in our cohort. Mean PCM area in the 40 PUM with M3 was significantly higher than in the 35 PUM with normal (disomy) chromosome 3 status (Figure 2A; *p* < 0.01). Although M3 PUM had an increased mitotic score overall (Figure 2B; *p* < 0.05), M3 was associated with significantly higher PCM area in the subgroup of PUM with low mitotic score (Figure 2C; *p* < 0.05), which exhibited similar mean PCM area to the high mitotic score tumours. Thus, centrosome aberrations correlate with M3, independent of mitotic score.

We also investigated potential correlation between other prognostic chromosomal alterations and centrosome aberrations. Gain of 8q in addition to M3 is a further indicator of poor outcome [5, 9, 10]. However, 8q gain was not associated with mean PCM area (Figure 2D), despite increased mitotic scores (Figure 2E; *p* < 0.01). In contrast to M3, when PUM was categorised into low or high mitotic index groups, 8q gain had no independent effect on mean PCM area in either group (Figure 2F), consistent with the idea that 8q gain alone is unlikely to be a driver of centrosome aberrations. Indeed, M3 PUM had significantly greater mean PCM area than disomy 3 PUM, irrespective of 8q status (Figure 2G; *p* < 0.05). Interestingly, PUM with gain of 6p, usually associated with good prognosis, had significantly lower mean PCM area than disomy 6 tumours (supplementary material, Figure S2A; *p* < 0.01) despite comparable mitotic scores (supplementary material, Figure S2B), further supporting the concept that centrosome aberrations are associated with aggressive disease and poor outcomes. In addition to these common chromosome alterations, tumour cell morphology is also used as a prognostic indicator in UM, with epithelioid‐celled tumours being associated with a poorer prognosis [41]. However, PUM classed as epithelioid during routine pathology did not show a significant difference in PCM size compared to predominantly spindle‐celled tumours (supplementary material, Figure S2C).

When patient follow‐up data were segregated by centrosome size, the cohort with larger centrosomes exhibited significantly worse survival (Figure 2H; *p* = 0.005). Taken together, our study of PUM reveals that UM cells display significant centrosome amplification compared to adjacent normal cells and that this is associated with M3, a major prognostic indicator in UM.

### Centrosome amplification is present in patient‐derived PUM cells and UM cell lines

**Figure 3:**
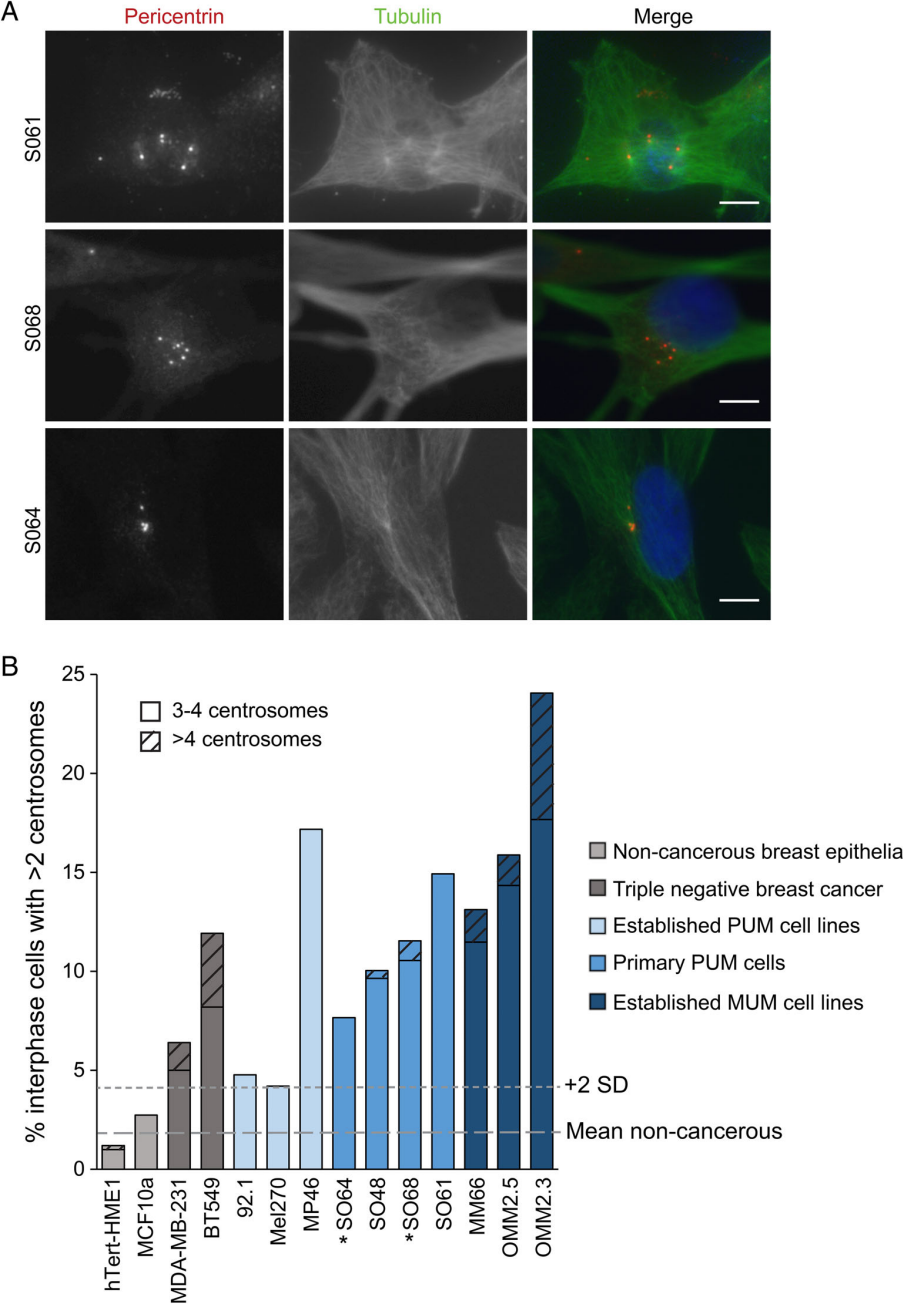
Interphase UM cell lines and patient‐derived cells exhibit centrosome amplification. (A) Examples of early passage cells derived from patients following enucleation with >2 centrosomes (pericentrin foci). Cells were fixed and stained with pericentrin (centrosome marker), tubulin (microtubules), and DAPI (DNA). Scale bars 10 μm. (B) Established UM cell lines and four patient‐derived PUM cell lines were stained with pericentrin, tubulin, and DAPI to score the percentage of interphase cells displaying more than two centrosomes. Grey lines indicate the mean and mean plus 2 standard deviations of the non‐cancerous cells (hTERT‐HME1 and MCF10A) used to define centrosome amplification. More severe instances of centrosome amplification (>4 centrosomes) are indicated by hatching. Number of interphase cells quantified: hTERT‐HME1: 504, MCF10A: 513, MDA‐MB‐231: 500, BT549: 537, Mel270: 596, 92.1: 42, OMM2.3: 736, OMM2.5: 649, MP46: 99, MM66: 122, SO64: 222, SO48: 249, SO68: 702, SO61: 67. *Monosomy 3 cells.

To allow more in‐depth analysis of centrosome amplification in UM, primary cultures derived from patients following enucleation and established UM cell lines (supplementary material, Table S1) were analysed by immunofluorescence staining. The number of pericentrin‐stained centrosomes in interphase cells were counted; cells with more than two centrosomes were considered to have centrosome amplification (Figure 3). In addition to UM cells, four cell lines of known centrosome status were included as controls. hTert‐HME1 and MCF10A cells are non‐cancerous breast epithelial cell lines that display low levels of centrosome amplification [42, 43]. Reflecting previous studies [29], we used the mean score plus two standard deviations for these non‐cancerous cells as the cut‐off to define whether other cells displayed centrosome amplification. MDA‐MB‐231 and BT549 cells are derived from triple‐negative breast cancer and several studies have demonstrated that these cell lines display marked centrosome amplification [18, 29, 31, 42, 44]; our data agree with these findings (Figure 3B). Importantly, all the patient‐derived PUM cells displayed levels of centrosome amplification that were comparable to, or higher than, that of the triple‐negative breast cancer cell lines (Figure 3B). These data support our earlier observations in fixed UM tissue sections (Figure 1). In addition, all the established UM cell lines showed centrosome amplification, with one cell line derived from a primary tumour and all three MUM cell lines displaying very high levels of centrosome amplification (Figure 3B). Indeed, the metastases‐derived cell lines also showed the highest prevalence of severe centrosome amplification, defined as having more than four centrosomes. Together, these data confirm that centrosome amplification is prevalent in UM cells, particularly in those derived from more aggressive forms of the disease.

### Comparison of patient‐matched UM cell lines shows a high level of centrosome amplification in metastatic cells

**Figure 4:**
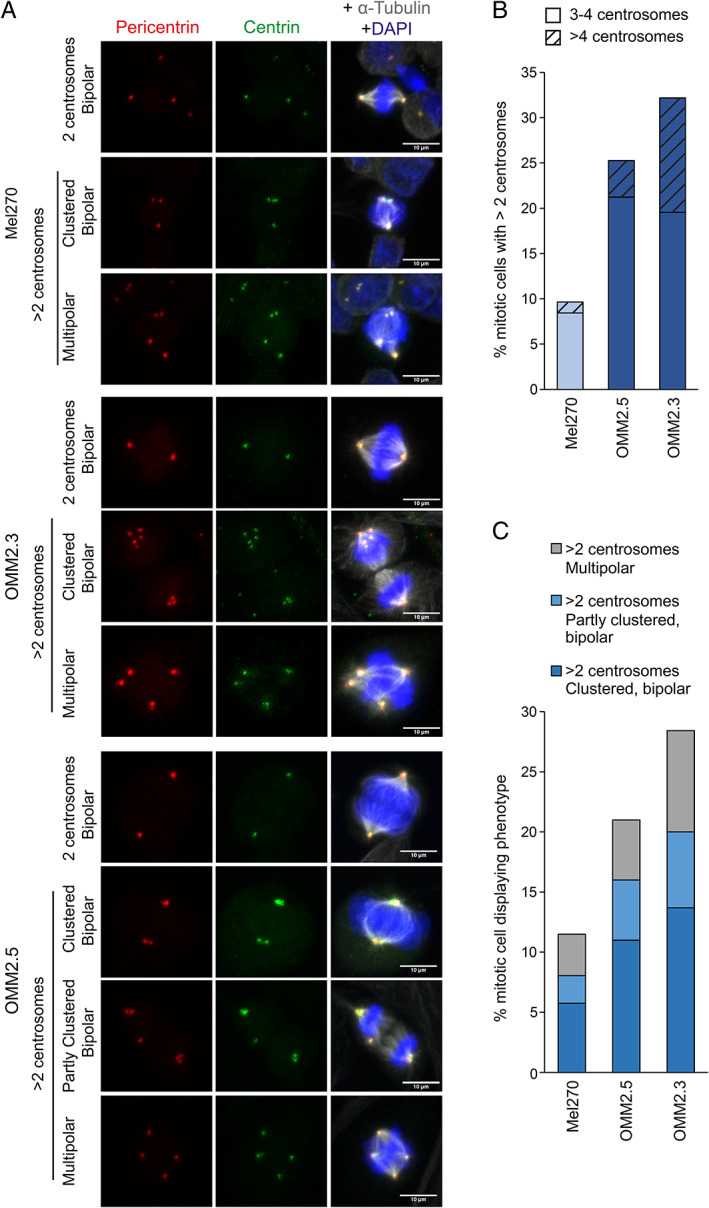
Detailed characterisation of patient‐matched UM cell lines reveals a high level of targetable centrosome amplification in MUM cells. The CA status for three patient‐matched cell lines was characterised in mitotic cells. Mel270 (derived from primary tumour), OMM2.5 and OMM2.3 cells (derived from distinct liver metastases of the Mel270 primary tumour) were fixed and stained with dual centrosome markers, pericentrin (PCM marker) and centrin (centriole marker), as well as alpha‐tubulin and DAPI. Samples were blinded and Z‐stacks covering the full depth of >85 individual mitotic cells for each cell line acquired. (A) Representative maximum‐intensity projections of Z‐stacks, showing observed phenotypes. Scale bars 10 μm. (B) Mitotic cells with >2 centrosomes were quantified. Cells with >4 centrosomes are indicated by hatching. Number of mitotic cells quantified Mel270: 87, OMM2.5: 100, OMM2.3: 95 (C) Mitotic spindle phenotypes of cells displaying centrosome amplification.

The differing centrosome amplification status of Mel270 (PUM) compared to OMM2.3 and OMM2.5 (MUM) cells was of particular interest as all three cell lines originate from the same patient. More in‐depth analysis was carried out using pericentrin and centrin to double label *bona fide* centrosomes (Figure 4A) and scoring centrosomes in mitotic cells that would normally have two centrosomes (Figure 4B), which allows more accurate quantification of centrosome amplification than interphase cells that may normally have either one or two centrosomes. These analyses corroborate centrosome amplification scores from interphase cells (Figure 3) but indicate a higher frequency of centrosome amplification, with 10% of Mel270, 25% of OMM2.5, and 32% of OMM2.3 exhibiting centrosome amplification. A particularly high proportion of OMM2.3 cells have extreme centrosome amplification, with more than 10% of cells harbouring more than four centrosomes (Figure 4B).

### Centrosome clustering and other coping mechanisms are evident in UM cell lines

Different mitotic phenotypes were observed amongst those UM cells displaying centrosome amplification, with some cells forming a multipolar mitosis and others clustering their supernumerary centrosomes to form a pseudo‐bipolar spindle (Figure 4A,C). Multipolar phenotypes occur when more than two nodes of centrosomes nucleate a mitotic spindle in a cell with centrosome amplification, resulting in an aberrant mitosis that is difficult for the cell to survive. A clustered pseudo‐bipolar mitosis occurs when a cell with centrosome amplification clusters the supernumerary centrosomes together so that cells can divide in a manner resembling a normal bipolar mitosis. Some cells with centrosome amplification exhibited partial clustering, forming a pseudo‐bipolar spindle, but also having additional centrosomes that were inactivated and not forming spindle fibres (Figure 4A). These cells are also partially reliant upon clustering to complete mitosis. Thus, despite their differing levels of centrosome amplification (Figure 4C), all three cell lines likely depend on centrosome clustering to some extent for their survival.

## Discussion

Here, we have demonstrated for the first time in UM patient samples, patient‐derived PUM cell cultures, as well as in established UM cell lines that centrosome amplification exists in a subset of UM, particularly in those tumours that are considered high risk for dissemination. Indeed, in PUM tissue samples, centrosome size correlates with patient survival. Centrosome amplification can induce oncogenic phenotypes such as aneuploidy and increased invasiveness [19, 21, 45], raising the possibility that it may be a driver in the development of MUM, and could offer an additional therapeutic angle.

Gene expression signatures have been used to predict centrosome amplification, including CA20, which is based on 20 genes that are either critical to centrosome structure or whose overexpression can induce centrosome amplification [46]. Computational analysis of the TCGA pan‐cancer data for the CA20 signature found high expression in many cancers, which was associated with genome instability and poor prognosis [47]. Whilst UM had a significantly lower overall CA20 score than skin melanoma in that study, a high CA20 score was associated with significantly worse survival in PUM [47]. This is in keeping with our findings that centrosome amplification is associated with M3 in PUM, the strongest single predictor of disease progression to metastasis and poor survival outcomes [4, 5] and that centrosome amplification is more extreme in cell lines derived from MUM.

Centrosome amplification drives low‐level aneuploidy [19], and this is an important factor in how it can drive tumourigenesis [48]. Although UM typically has a defined genetic profile, with quite limited karyotypic changes, M3 tumours do show increased aneuploidy compared to disomy 3 UM [49], consistent with our observation that M3 UM display increased centrosome abnormalities.

The tumour suppressor BRCA1‐associated protein‐1 (BAP1) is a nuclear deubiquitylase encoded on chromosome 3p. BAP1 loss and functional inactivation are strongly associated with M3, and with UM metastasis and poor prognosis [9, 50, 51]. In addition to the association between M3 and centrosome amplification in PUM, in our cell studies, MP46, a cell line which is nuclear BAP1 negative [52], displayed one of the highest levels of centrosome amplification. Whilst BAP1 loss is uncommon in skin melanoma, it is associated with a subset of cutaneous non‐melanoma tumours called BAP1‐inactivated melanocytic tumours (BIMTs) [53]. It was recently reported in a survey of BIMTs from 10 patients that these show reduced ciliation and increased centrosome amplification compared to conventional melanocytic nevi [54]. Mechanistically, BAP1 has been found to deubiquitylate both gamma‐tubulin in breast cancer [55] and the centrosome protein MCRS1 in renal cell carcinoma [56], affecting the mitotic spindle and centrosome formation. Together with our findings, these studies raise the intriguing possibility that BAP1 loss could play a more general role in centrosome amplification in cancer, which will merit future investigations.

Whilst PUM is successfully locally controlled through surgical resection and/or radiotherapy [12, 13], MUM, which typically arises in the liver, is associated with a poor outcome [16, 17]. We show that aggressive subtypes of PUM samples and MUM cell lines display a high degree of centrosome amplification and that UM cells often utilise centrosome clustering to allow them to undergo mitosis suggesting the potential for therapeutic intervention. The concept of targeting centrosome clustering as a specific anti‐cancer treatment is well established in the literature [31, 32, 45, 46, 57, 58] with ongoing studies exploring therapeutically relevant tools to disrupt clustering, whilst disruption of alternative coping mechanisms such as centrosome inactivation, which we also observed in MUM cells (Figure 4A), or even reversing centrosome amplification to temper oncogenic phenotypes and metastatic potential [33] may also be explored. As outcomes for MUM are particularly poor, such strategies for targeting centrosome amplification are valuable directions for future research.

In summary, our work provides the foundations for future research on centrosome amplification as a potential driver and therapeutic target in UM, which currently lacks effective drugs for curing metastatic disease.

## Author contributions statement

DS‐P and KF‐K contributed equally to this study; they carried out the experiments, collected and analysed the data and generated the figures. NK carried out the experiments and collected the data. SEC and HK conceived the experiments and interpreted the data. JMC conceived the experiments, interpreted the data and wrote the manuscript. ABF conceived the experiments, analysed the data, generated the figures and wrote the manuscript. All authors were involved in writing the paper and had final approval of the submitted and published versions.

## Supporting information


**Figure S1.** Mitotic score is positively correlated with the number of Ki67‐positive cells and PCM size in UM samples
**Figure S2.** Mean PCM area is associated with chromosome 6p status but not with cellular morphology in PUM samples
**Table S1.** UM cell line characteristics
