# Supplementary material for: Aggressive uveal melanoma displays a high degree of centrosome amplification, opening the door to therapeutic intervention
Source: J Pathol Clin Res. 2022 Apr 26;8(4):383–94. doi: 10.1002/cjp2.272 (PMC9161346; doi:10.1002/cjp2.272)
Supplement: Supplementary file 1 — Figure S1. Mitotic score is positively correlated with the number of Ki67‐positive cells and PCM size in UM samples Figure S2. Mean PCM area is associated with chromosome 6p status but not with cellular morphology in PUM samples Table S1. UM cell line characteristics [file CJP2-8-383-s001.pdf]

Aggressive uveal melanoma displays a high degree of centrosome amplification, opening the door to therapeutic intervention

D Sabat-Pośpiech, K Fabian-Kolpanowicz *et al*. *J Pathol Clin Res* DOI: 10.1002/cjp2.272

Supplementary Figures S1 and S2

Supplementary Table S1

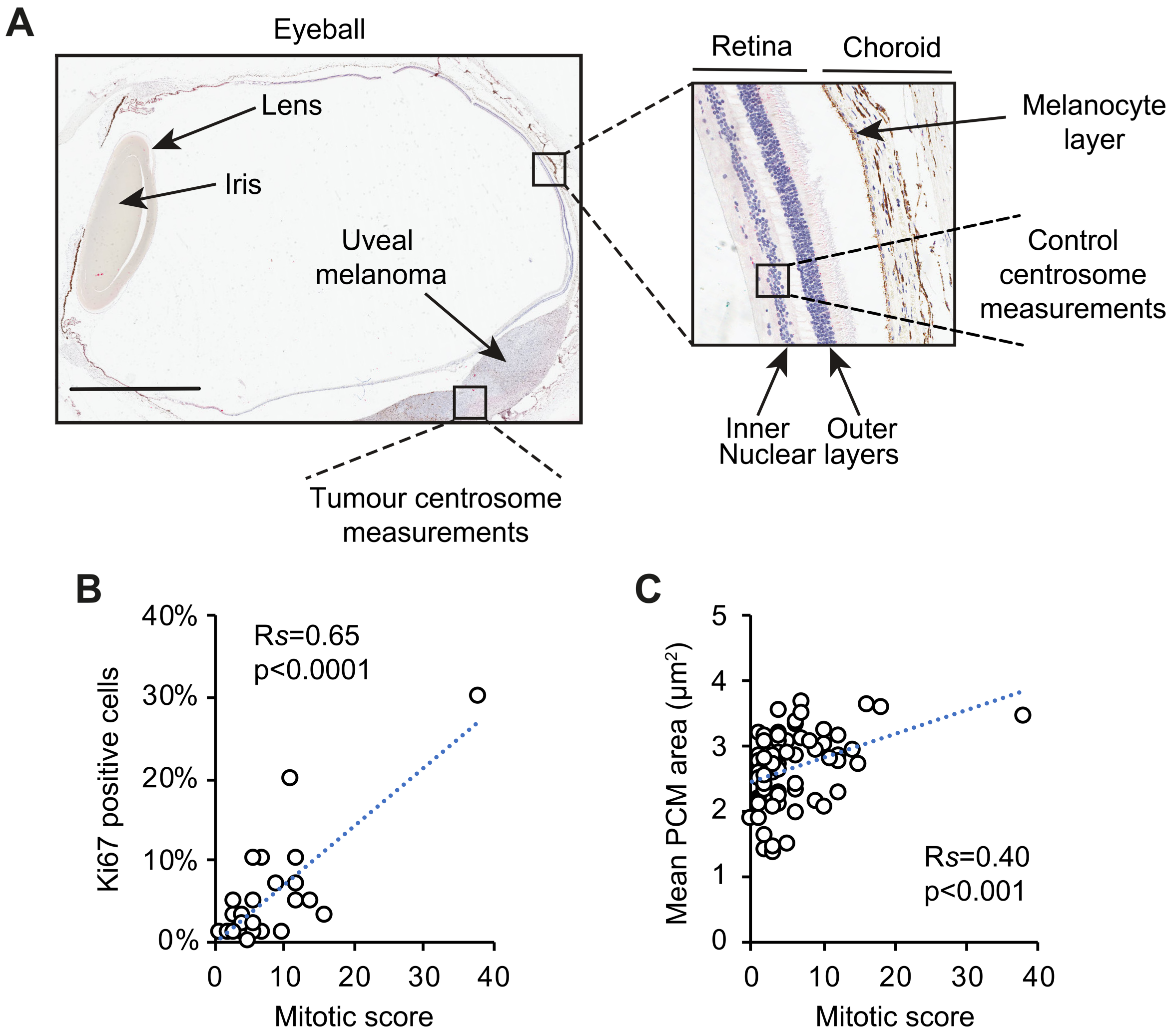

Figure S1. Mitotic score is positively correlated with number of Ki67 positive cells and pericentriolar matrix size in UM samples.

(A) An example image of the uvea with the regions used for centrosome amplification measurements indicated. Scale bar 5mm. **(B, C)** Mitotic score was calculated as number of mitoses per 40x field of view. Blue dashed trendline includes all values; statistical significance tested using one-tailed Spearman correlation coefficient. **(B)** Scatter plot comparing the mean number of Ki67 positive cells with mitotic score. **(C)** Scatter plot comparing the mean PCM size with mitotic score.

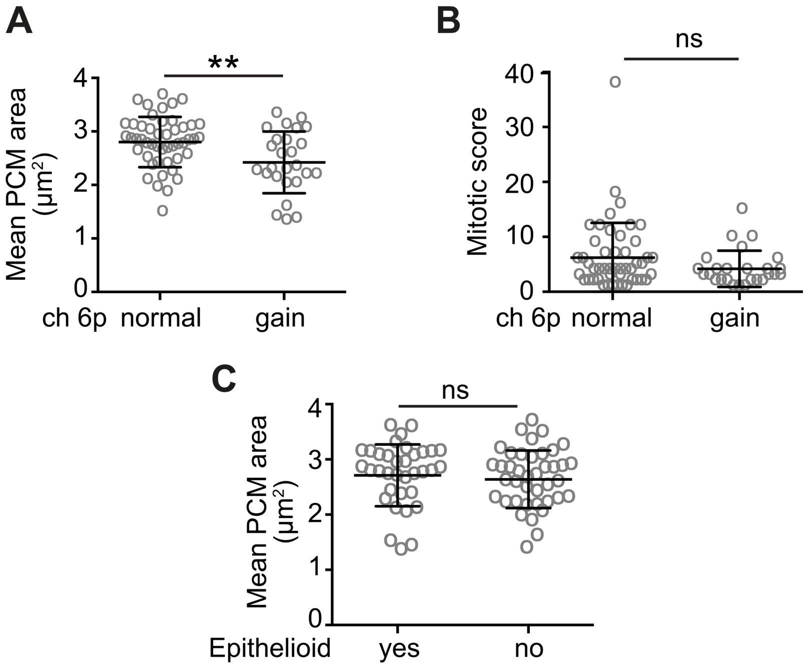

Figure S2. Mean PCM area is associated with chromosome 6p status but not with cellular morphology in PUM samples.

(A & B) Comparison of **(A)** mean PCM size and **(B)** mitotic score in UM tumours with different chromosome 6p status: normal (n=48) versus gain (n=26). Unpaired two-tailed T test, ns – not significant, **p<0.01. (**C**) Comparison of mean PCM size in UM tumours categorised by different cell shape: epithelioid (n=35) versus non epithelioid (n=40). Unpaired two-tailed T test, ns – not significant.

Cellular characteristics, protein expression and genetic alterations	Uveal Melanoma cell lines					
	92.1	MP46	Mel270	Omm2.5	Omm2.3	MM66
Origin	Primary	Primary	Primary	Liver Metastasis	Liver Metastasis	Liver Metastasis
Cell Type	Epithelioid	Mixed (Spindle and Epithelioid)	Spindle	Mixed (Spindle and Epithelioid)	Mixed (Spindle and Epithelioid)	Mixed (Spindle and Epithelioid)
MelanA	+	+	+	+	+	+
nuclear BAP1 (IHC)	+	-	+	+	+	+
Chr 3	Partial losses	Loss	Normal	Normal	ND	Normal
Chr 8q	Gain	Gain	Normal	Normal	ND	Gain
GNAQ mutation	c.626 A>T	c.626 A>T	c.626 A>C	c.626 A>C	c.626 A>C	-
GNA11 mutation	-	-	-	-	-	c.626A >T
BAP1 mutation	ND	-	ND	ND	ND	-
SF3B1 mutation	-	-	-	-	ND	-
EIF1AX mutation	c.17 G/A	-	-	-	ND	-

Table S1. UM cell line characteristics.

ND, not determined; IHC, immunohistochemistry
